# DNA damage induced by ZnO and CuO nanoparticles: a comparative study against bulk materials

**DOI:** 10.1007/s11356-025-36726-4

**Published:** 2025-07-24

**Authors:** Paulina Abrica-González, Sandra Gómez-Arroyo, Antonio Sotelo-López, Aron Jazcilevich-Diamant, Ana Rosa Flores-Márquez, Josefina Cortés-Eslava

**Affiliations:** 1 Laboratorio de Genotoxicología Ambiental, Instituto de Ciencias de la Atmósfera y Cambio Climático, Universidad Nacional Autónoma de México, Ciudad Universitaria, Coyoacán, Mexico City, 04510 Mexico; 2 Departamento de Ingeniería en Comunicaciones y Electrónica, Escuela Superior de Ingeniería Mecánica y Eléctrica Unidad Zacatenco, Instituto Politécnico Nacional, Gustavo A. Madero, Mexico City, 07738 Mexico; 3 Grupo de Fisicoquímica Atmosférica, Instituto de Ciencias de la Atmósfera y Cambio Climático, Universidad Nacional Autónoma de México, Ciudad Universitaria, Coyoacán, Mexico City, 04510 Mexico

**Keywords:** Atmospheric pollution, Bioindicators, Comet assay, *Ricinus communis*, Toxicity

## Abstract

As nanoparticles are increasingly incorporated into consumer products and specialized areas, the need to study their toxicity and the importance of caution in their use are becoming more evident. The use of these novel materials in the automotive industry is causing increased nanoparticle emissions into the atmosphere, sparking concerns due to recent toxicology reports. Detecting and characterizing nanostructured atmospheric pollutants represent a complex task that alternative methods like genotoxic bioindicators may overcome. *Ricinus communis* was selected as a model plant to describe the genotoxic effect of air-dispersed nanostructured materials, using comet assay to evaluate the DNA damage by exposure to ZnO and CuO nanoparticles (ZnO-NPs, CuO-NPs), and total chlorophyll was determined to correlate the results. The plants were exposed for 1.25 h by nebulizing dispersions of the nanoparticles, and their effects were evaluated at 24 h and 5 days after exposure. Time was essential in demonstrating the DNA damage induced by lower levels of CuO-NPs and ZnO-NPs, which showed more significant damage after 5 days than bulk materials. In both compounds (CuO and ZnO), significant differences were observed in the comparison of their nanoscale and their bulk form, which confirms that the lower size of the nanoparticles presents a more significant genotoxic potential. In the scanning electron microscopy (SEM) micrographs, the accumulation of nanoparticles was observed near the stomata. The study demonstrated the viability of the comet assay as a sensitive tool both to assess DNA damage from ZnO-NPs and CuO-NPs on *R. communis* as a bioindicator and to investigate the potential risks of emerging nanomaterials, emphasizing the need for caution in their use.

## Introduction

Air pollution is a significant issue caused by various natural and anthropogenic sources, including the automotive sector (Dong et al. 2021). While the industry is seeking low-cost, efficient, and sustainable materials (Ibrahim et al. 2016) that can leverage various thermal, optical, magnetic, and electrical properties (Abrica-González et al. 2018), it is important to ensure that the use of these materials does not contribute to additional pollution.

As such, researchers are studying the environmental impact of these new nanomaterials, including potential emissions during production and disposal (Maynard et al. 2006). Metal oxides like MnO, ZnO, CuO, and TiO_2_ are among the most commonly used additives that can potentially reduce the environmental impact of the automotive industry by improving fuel efficiency and reducing emissions (Yuvarajan et al. 2018). However, their environmental impact must be closely examined through careful evaluation and monitoring due to the possible presence of emerging contaminating materials such as nanoparticles. The presence of metallic nanoparticles in the atmosphere has increased mainly due to the automotive sector (Rabajczyk et al. 2020; Rai 2016). The development of new nanostructures is continually increasing both the quantity and diversity of nanoparticles in the environment, potentially leading to severe environmental consequences if left unchecked (Calderón-Garcidueñas et al. 2022).

While toxicological reports on these materials are available, certain ones have found utility in agricultural practices, such as their application as fertilizers. These substances exhibit the potential to enhance growth and nitrogen fixation. However, they come with inherent drawbacks, including their tendency to bioaccumulate within seeds and leaves (Siddiqi and Husen 2017), resulting in adverse impacts on germination (Lee et al. 2010). Furthermore, their possible internalization through stomata has been observed (Lv et al. 2019). Considering this, we deemed it essential to delve into the genotoxic effects of ZnO and CuO on plants following leaf exposure via nebulization, a process where a fine mist of the nanoparticle solution is sprayed onto the plant leaves. This approach was selected to emulate the naturally occurring way of leaf exposure to airborne nanoparticulate contaminants, as these pollutants could plausibly be conveyed through droplets originating from air humidity and mist.

Using plants as bioindicators has emerged as an effective complementary tool for assessing the genotoxic potential of environmental contaminants. The selection of the best plant species is a critical challenge, as different plant species respond differently to environmental pollutants (Khalid et al. 2019). Plants not only enable the detection of harmful concentrations of pollutants in soil and air but also provide valuable insights into changes in the bioavailability and behavior of these compounds within plant tissues. The attributes that make plants excellent biomonitors include their high sensitivity to pollutants, bioaccumulation capacity, and most importantly, their rapid growth and reproduction. These last highlight the urgency of our work in understanding and mitigating the effects of environmental contaminants, and the ability to express various biomarkers that reflect exposure to and the effects of toxic agents (Rai 2016; Asif et al. 2018; Świslowski et al. 2022). The fundamental challenge that highlights the significance of our work is identifying the best plant species to use because different plant species respond differently to environmental pollutants (Khalid et al. 2019).

Several plant species, including *Taraxacum officinale*, *Lepidium sativum*, *Arabidopsis thaliana*, and *Medicago sativa*, have been utilized as model organisms to assess the toxicological impact of nanoparticles (Slomberg and Schoenfisch 2012; Raklami et al. 2021; Abrica-González et al. 2023; Mosenoka et al. 2024). *Ricinus communis*, a potential game-charger in the field, is reported to be an effective biomonitoring plant (Mendes et al. 2015; Khalid et al. 2019), but its response to nanoparticulate atmospheric emissions has not been investigated previously. *R. communis* is a very adapted exotic invasive species with many applications, including traditional medicine and oil production (Lomash et al. 2010). It has even been used to synthesize nanoparticles to test its activity against both microbial pathogens and cancer cell lines, with interest in the potential use in drug and DNA delivery (Abdul et al. 2018). The plant seeds have been tested as indicators of tolerance to Cd and Pb and can be potentially used for phytoremediation of contaminated areas (de Souza Costa et al. 2012).

Part of the success of this plant is due to its high tolerance to highly contaminant environments, like in urban areas, where vehicle traffic has become the most significant source of air pollution. It is the main factor contributing to the bad air quality in cities, where exposure to particles with diameters below 1 mm is raising concerns, as they are more easily incorporated through respiration (Dailey et al. 2003). *R. communis* is a good bioindicator of genotoxicity and bioaccumulation near roadsides because it is a significant plant species from an environmental perspective (Khalid et al. 2019). Furthermore, plants have been shown to provide adequate results for various environmental parameters, such as air pollution and soil contamination (Baker et al. 1994).

Notably, it is essential to find potential plant species that can be used to evaluate the status of bad air quality and trends related to the health of humans. However, it is important to consider that the results may vary depending on the type of pollutant, and careful consideration should be given to selecting appropriate plants for a given situation (Muthusaravanan et al. 2018).

The alkaline comet assay introduced by Singh et al. (1988) is a versatile and highly sensitive technique to evaluate genotoxic damage in individual cells. As plant cells have a wall, Gichner and Plewa (1998) developed a very efficient mechanical method for isolating nuclei from the leaves of *Nicotiana tabacum.* Evaluating genotoxicity in plants using the comet assay is a useful complement to conventional phytotoxicity indicators such as chlorophyll content. Its ability to detect DNA strand breaks and oxidative damage makes it a reliable and cost-effective technique for assessing the effects of nanomaterials and environmental pollutants, thereby enhancing the practicality of ecotoxicological research. Its use has expanded notably in ecotoxicological research, particularly in studies involving nanoparticles and metal oxides, with plant species like *Taraxacum officinale* proving to be effective models (Cortés-Eslava et al. 2018). Considering the above mentioned, the aim of this work is to evaluate *Ricinus communis* as a bioindicator of potential contaminants in urban areas (CuO-NPs and ZnO-NPs), evaluate its toxic and genotoxic effects with the comet assay on these plants, and analyze its possible bioaccumulation inside its leaves.

## Materials and methods

### Sources of chemicals

Copper oxide nanopowder (< 50 nm, Prod. No. 544868), zinc oxide nanopowder (< 50 nm, Prod. No. 677450), copper oxide powder (< 10 µm, 98%), zinc oxide powder (< 5 μm particle size), ethidium bromide, low melting point agarose (LMPA), potassium dichromate, and sodium hydroxide were acquired from Sigma Aldrich (Mexico). Normal point melting agarose (NMPA), ethylenediaminetetraacetic acid (EDTA), and tris(hydroxymethyl)aminomethane were purchased from Invitrogen (Mexico) ultraPURE. All CuO and ZnO nanoparticles and bulk powders were diluted in distilled water, and the solutions were sonicated for 2 h before each experiment. All glassware was thoroughly washed with aqua regia and rinsed with Milli-Q water to avoid metal contamination before use.

### Size and ζ-potential of nanoparticles

The morphology of ZnO-NPs and CuO-NPs was characterized by atomic force microscopy (AFM) (Innova, Bruker); samples were prepared by dispersing the solutions in alcohol at a concentration of 1 ppm and left to dry over a glass substrate adhered to the microscope stub with double-sided carbon tape. SEM micrographs were used to obtain the size distribution histograms; nanopowders were mounted on SEM stubs with double-sided carbon tape, and micrographs were obtained with an accelerating voltage of 20 kV.

For UV–vis absorption spectroscopy, both studied nanoparticles were dispersed in Milli-Q water at a concentration of 50 mg/L and sonicated for 30 min; samples were processed in a Cintra 1010 (GBC Scientific Equipment) with standard 10 mm quartz cells. Optical properties: Zeta potential, hydrodynamic diameter, and polydispersity index (PDI) were obtained with a Malvern Zetasizer Nanoseries (Malvern, UK) at room temperature, using DTS1060 capillary cells (Malvern Panalytical Ltd).

### Plants exposure to nanoparticles

**Fig. 1:**
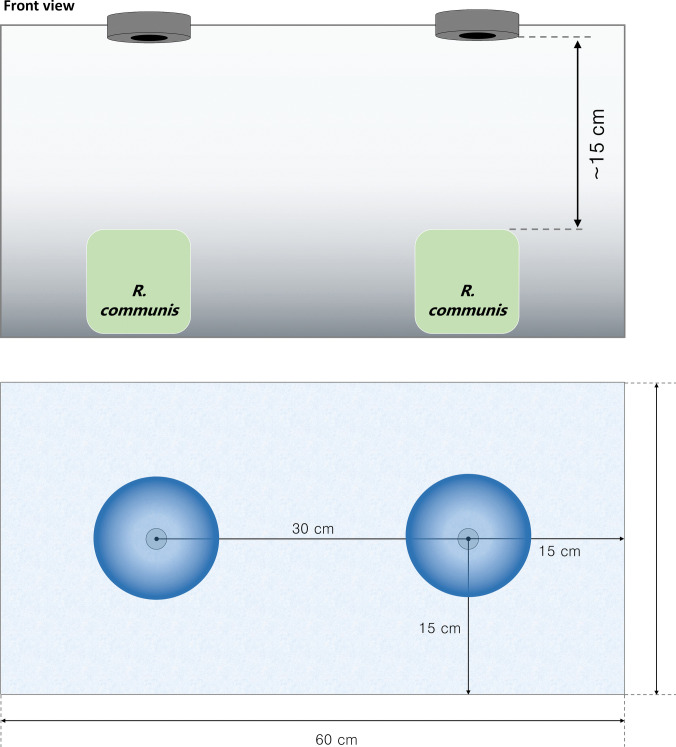
Nebulization chamber design. Two nebulizers were adapted at the top of the PMMA box

The utmost care was taken in the plant exposure process, including the use of 115 kHz ultrasonic piezoelectric ceramic nebulizers (Zibo Yuhai Electronic Ceramic, China) and the adaptation of the nebulizers to the cap of 100 mL glass vials to ensure the accuracy of our results. The outlet of nebulizers was placed directly over the perforations at the top of custom-built nebulization chambers with dimensions of 600 × 300 × 400 mm, allowing the exposure of two plants per box (Fig. 1). *R. communis* secondary branches were cut by the stem (15 cm from the stem to the leaf, three leaves by stem) from healthy wild plants and sent immediately for adaptation to laboratory conditions according to the described by Misik et al. (2011). To preserve the physiological state of the cuts, they were kept under controlled conditions of light and temperature, and the water was maintained with constant aeration to avoid stressing them. Stems were placed in glass vials with tap water for 24 h, with 12 h light/12 h dark at room temperature before starting treatment inside the chamber. Plant exposure was performed by nebulizing the prepared dispersions of CuO-NPs and ZnO-NPs in distilled water. Test treatments (Bulk ZnO, CuO, and ZnO-NPs, and CuO-NPs) were dispersed in distilled water at 50, 100, 200, and 800 mg/L concentrations and sonicated before nebulization. Studies previously conducted on the application of CuO and ZnO nanoparticles in plants served as the basis for selecting the concentrations. Distilled water and potassium dichromate solution (0.05 M) were used as negative and positive controls, respectively. The outflux of each nebulizer was assessed, resulting in a steady flow of 0.04 L/H consisting of 5 µm droplets that form a cone of 20°. A single exposure was performed for each concentration and type of compound. Each exposure was performed with 50 mL of treatment dispersion per vial, nebulizing a total of 100 mL within the 72 L chamber; treatments were administered for 1.25 h each. Three independent repetitions were conducted, each using a different pair of plants for every concentration tested. Following each exposure cycle, the chamber remained sealed for about 2 h to allow the mist to settle. At the end of each cycle, the chambers were thoroughly cleaned. All plants were maintained in water with continuous aeration until used in the respective experiments. A datalogger (CR1000, Campbell Scientific), with humidity, temperature, and pressure transducers, was used to register the atmospheric data inside the chamber. Distinct chambers were dedicated to each treatment type (copper, zinc and both controls), maintaining a controlled and isolated experimental environment. The plants were analyzed 24 h and 5 days after exposure.

### Comet assay

The comet assay was performed under dim light conditions to minimize the potential influence of light on the results. All solutions, instruments, and glassware were stored at 4 °C before use and kept on ice during the procedure. Nuclei from 0.5 × 4 cm sections of plant leaves were extracted by making perpendicular cuts concerning the primary vein with a cold razor blade inside tilted Petri dishes so that the nuclei could be collected by precipitation in the cooled buffer solution (350 μL of 0.4 M Tris buffer, pH 7.5). This thorough procedure ensures the validity of the genotoxicity assessment, so that 50 μL of the nuclei suspension was added to 50 μL of 1% low melting point agarose (LMPA) at 40 °C, and was gently mixed. Next, 75 μL of the mixture was placed on a coverslip and immediately overlapped onto the microscope slide, previously covered with 0.5% normal melting point agarose, ensuring the avoidance of any bubble formation. The coverslip was carefully removed after placing the slide on a cold surface for 5 min for the agarose to solidify. Finally, the preparation was covered with a protective layer of 75 μL of LMPA at 37 °C (Gichner 2003).

The slides were incubated in electrophoresis buffer (300 mM NaOH, 1 mM EDTA; pH > 13) at 4 °C for 15 min to unwind DNA, followed by electrophoresis at 0.7 V/cm (25 V, 300 mA) for 20 min. Afterward, the slides were neutralized by rinsing with Tris 0.4 M three times for 5 min each, then fixation in pure ethanol for 5 min. The slides were then left to dry overnight. Staining was performed using ethidium bromide (20 μg/mL). The nuclei were observed under an epifluorescence microscope (Axiostar Plus Carl Zeiss) with an excitation filter of 515–560 nm and a barrier filter of 590 nm. At least 50 nuclei per slide and three slides were analyzed for each experiment. Image analysis was conducted using Comet Assay IV software, recording the tail length (µm), tail intensity (% of DNA in comet tail), and tail moment parameters.

### SEM micrographs

Following plant exposure, the presence of nanoparticles on leaf surfaces was examined using low vacuum scanning electron microscopy (LV-SEM) (Quanta 250 FEG, FEI Thermo Fisher Scientific) mounting fresh cuts of leaves (previously washed with running water and sonicated for 5 min) in SEM stubs with double-sided carbon tape. The obtained micrographs were carried out at a pressure of 0.4 to 0.5 mbar, an accelerating voltage of 15 kV, and a working distance of 10.5 mm. Elemental analysis was obtained with the SEM EDS (energy dispersive X-ray spectroscopy). One advantage of using low vacuum scanning electron microscopy (LV-SEM) is that the samples remain unaltered during preparation, and the vacuum does not affect the surface morphology of the leaves.

### Spectrophotometry analysis

To obtain the chlorophyll content per weight, we used 90% acetone to extract chlorophyll from the plant leaves five days after exposure and measured the absorbance of the extracts at 664.5 nm and 647 nm using VE 722–2000, Velab. The solid content was weighed after drying the same leaves used for extraction. Finally, we calculated the chlorophyll content using Eq. 1:

1 $$Totalchl=17.9\;A_{647}+8.08\;A_{664.5}$$

Chlorophyll quantification results were statistically analyzed by two-way ANOVA, comparing concentrations within each pair of treatments, ZnO vs ZnO-NPs, and CuO vs CuO-NPs.

### Statistical analysis

A statistical analysis of total chlorophyll content was performed using two-way ANOVA, considering the effects of concentrations and treatment. Three-way ANOVA was employed to compare the genotoxic effect of each pair of ZnO and CuO treatment sets in both bulk and nano form. The treatments (ZnO vs ZnO-NPs or CuO vs CuO-NPs), concentrations (negative control, 50, 100, 200, and 800 mg/L, and positive control), and time (24 h after treatment and 5 days after treatment) were treated as factors. A statistically significant result was considered when *p* < 0.05.

## Results and discussion

### Size and ζ-potential of nanoparticles

**Fig. 2:**
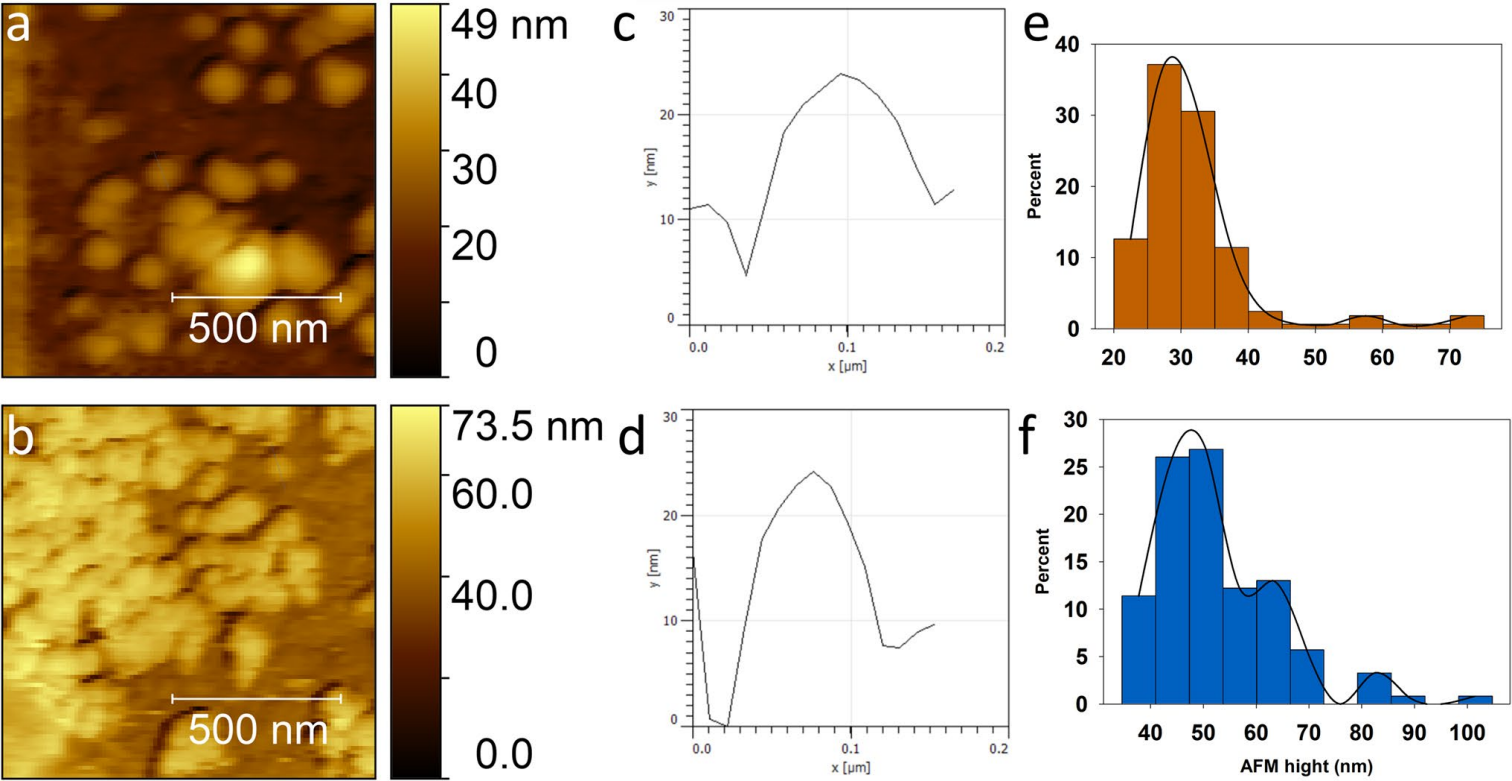
AFM micrographs of CuO-NPs (**a**) and ZnO-NPs samples (**b**), corresponding height profiles of representative particles (**c**), (**d**), and histogram of frequencies (**e**), **f**, respectively

The AFM data was analyzed to determine nanoparticle diameter by measuring the particle’s height peaks against the substrate. The mean diameter of CuO-NPs was found to be 31.986 ± 9.172 nm and 52.666 ± 11.823 nm for ZnO-NPs. Figure 2 shows AFM micrographs of CuO-NPs (Fig. a) and ZnO-NPs samples (Fig. 2b), along with the corresponding height profiles of representative particles (Fig. 2c, d) and histogram of frequencies (Fig. 2e, f), respectively. The measured diameters of both types of nanoparticles align with the nominal diameter reported by the manufacturer, which is < 50 nm.

**Fig. 3:**
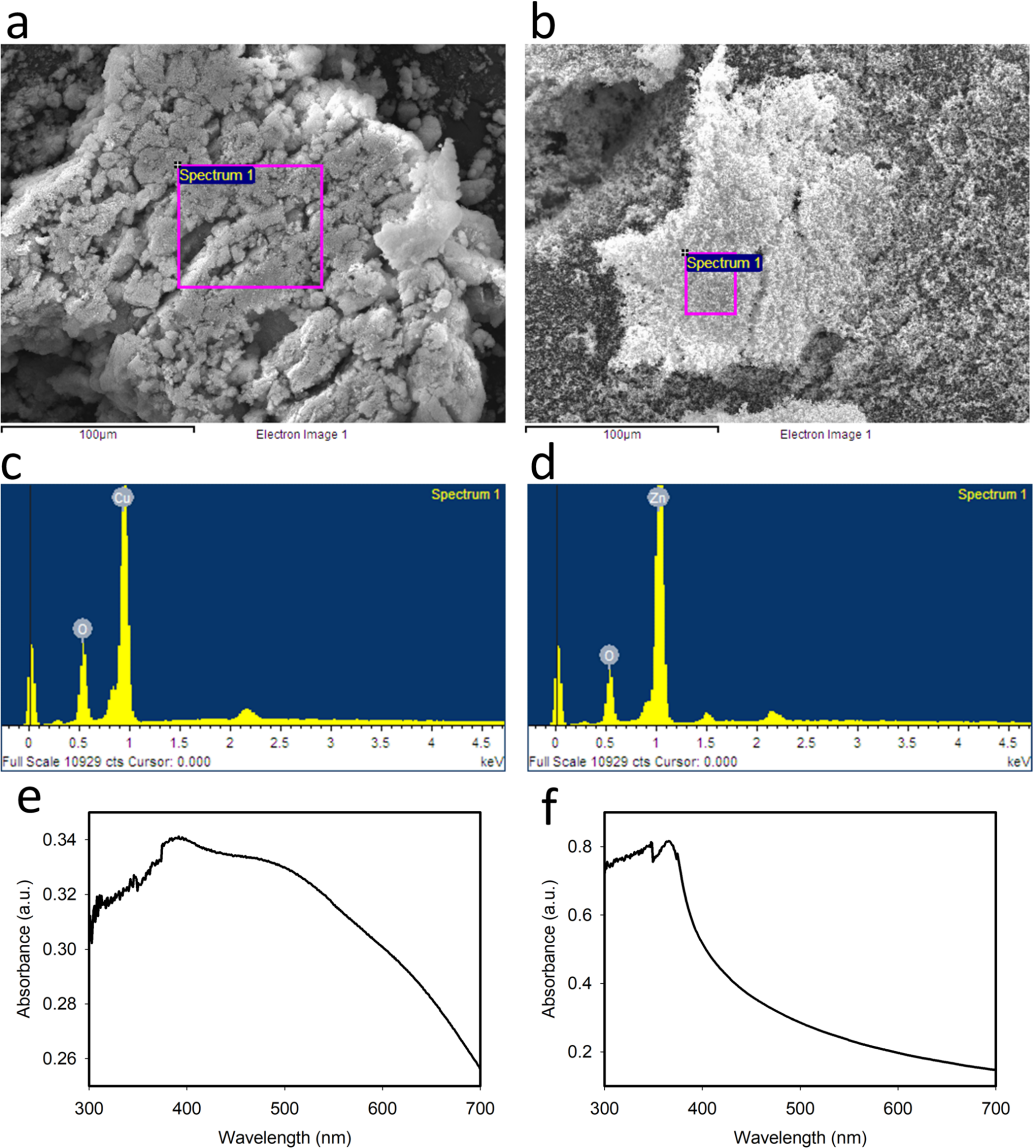
Elemental analysis and SEM micrographs (insets) of ZnO and CuO nanoparticles accumulated inside the stomata

Figure 3 shows the SEM micrographs of both studied nanoparticles CuO-NPs (Fig. 3a) and ZnO-NPs (Fig. 3b) with their respective elemental analysis (Fig. 3c, d), exhibiting the peaks corresponding to Cu and Zn.

The UV–Vis spectrum of CuO-NPs (Fig. 3e) exhibits a characteristic peak at 391 nm, which confirms the presence of CuO-NPs (Navada et al. 2020; Sharaf Zeebaree et al. 2021), and the UV–Vis spectrum of ZnO-NPs, which was scanned from 300 to 700 nm (Fig. 5f), exhibits a characteristic peak at 365 nm. This peak corresponds to the characteristic absorption of ZnO-NPs, and it is blue-shifted from the absorption peak of bulk ZnO at 376 nm (Miri et al. 2019). The mean hydrodynamic diameter value for CuO-NPs was 239.20 nm and for ZnO-NPs 251.19 nm with a polydispersity index of 0.341 and 0.381, respectively. The mean zeta potential of CuO-NPs at − 17.6 mV suggests better stability than ZnO-NPs, which presented a mean zeta potential of + 7.44 mV (Hou et al. 2017; Nabila and Kannabiran 2018). Previous reports suggest nanoparticle stability as indicated by the zeta potential is closely related to toxicity (Lv et al. 2019; Rotini et al. 2017). Specifically, higher zeta potential values, such as those found for ZnO-NPs, are often associated with greater toxicity due to increased particle aggregation and potential cellular uptake.

### Comet assay

**Fig. 4:**
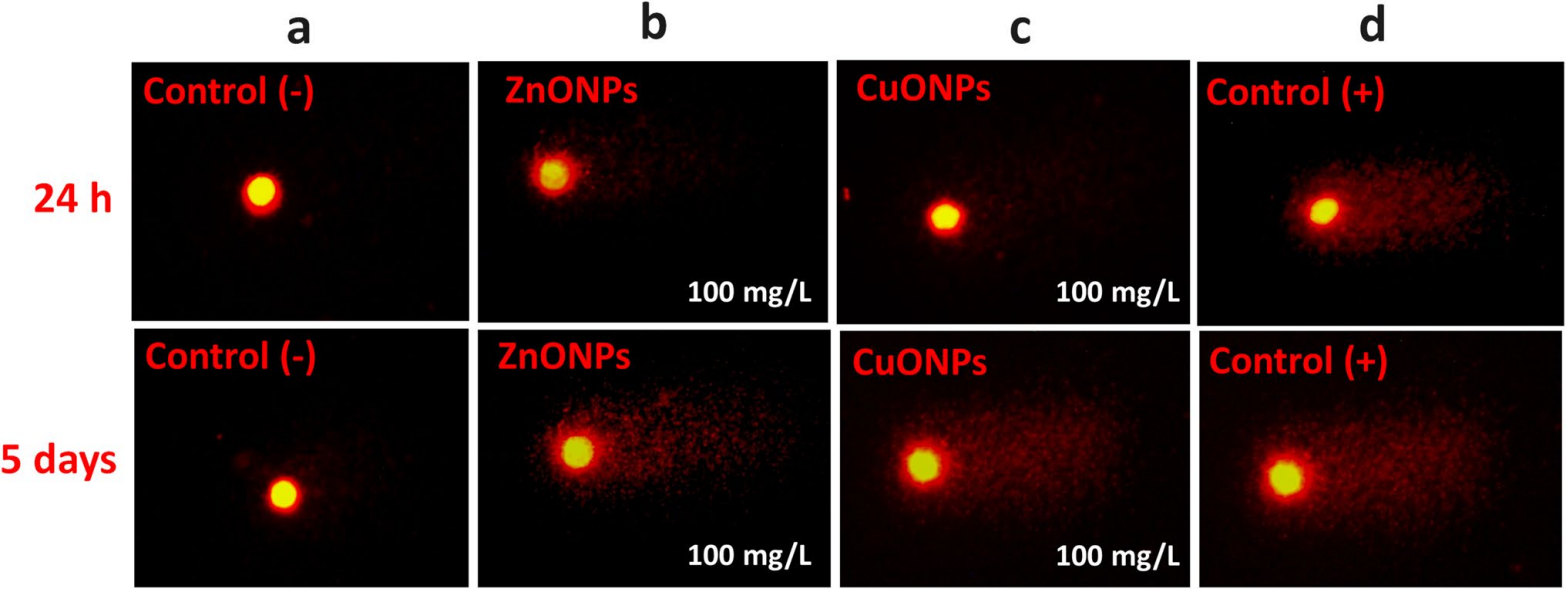
Summary of representative comets images at 100 mg/L against controls. Negative control (**a**), ZnO-NPs (**b**), CuO-NPs (**c**), and positive control (**d**); 24 and 5 days after exposure

To determine the genotoxic potential of CuO and ZnO nanoparticles, as well as their bulk counterparts, the alkaline comet assay was employed for detecting DNA damage, particularly strand breaks and other lesions that are converted into strand breaks under alkaline conditions. To achieve a rigorous quantification of the extent of induced genetic damage, the following analytical parameters were selected: tail length, tail intensity, and tail moment, in accordance with the methodological guidelines described by Koppen et al. (2017), Møller et al. (2020), and Collins et al. (2023). Figure 4 displays representative comet assay images illustrating the extent of DNA damage observed at a 100 mg/L concentration (central row), including negative and positive control (leftmost and rightmost columns, respectively). The high DNA damage at 100 mg/L for ZnO-NPs and CuO-NPs, like that of the positive control, is visually evident and is confirmed by the values obtained by digital image analysis. This visual confirmation highlights the significant relevance of the findings.

**Fig. 5:**
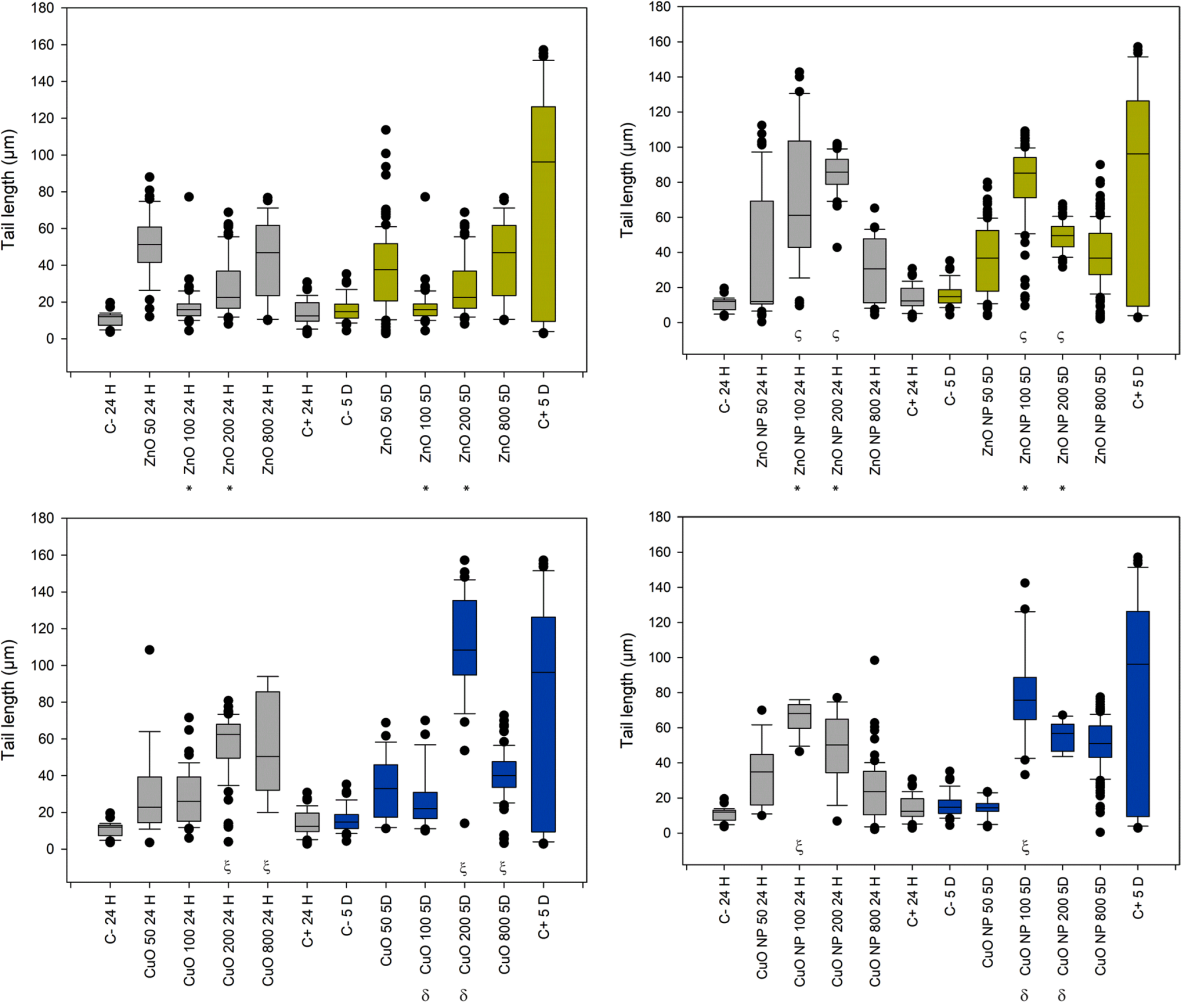
Box plots for the distribution of tail length results in *R. communis* exposed to ZnO and CuO (bulk and nano forms) after 24 h and 5 days of treatment. Negative control (C-), positive control (C+). The symbol ξ indicates a significant difference against negative control, within a specific time and treatment. The symbol δ indicates difference against negative control, within a specific time and treatment. The symbol ς indicates significant difference between bulk and nano treatments within concentration and time. The symbol * indicates a significant difference between bulk and nano treatments within concentration without considering the effect of time

Figure 5 summarizes the tail length results in plants exposed to ZnO and CuO compounds. The three-way ANOVA for ZnO treatments (bulk and nano) did not show an interaction among all factors; however, a significant interaction was observed between treatment and concentration, as well as between concentration and time. No concentration of bulk ZnO induced significant damage compared to the negative control. In contrast, 100 and 200 mg/L of ZnO-NP caused significant damage relative to the negative control, without considering the effect of time due to the absence of a three-way interaction among the factors.

The three-way ANOVA for the tail length of CuO data presented a triple interaction of factors, allowing the consideration of the effect of time. CuO-bulk treatment after 24 h presented a significant difference against negative control for 200 and 800 mg/L concentrations, and CuO-NPs presented a significant difference only at 100 mg/L. However, there is no statistically significant difference between the treatments within this time value. After 5 days of treatment, a significant difference can be found between the bulk and nano forms of CuO treatments, with a higher effect for CuO-bulk at 200 mg/L and 100 mg/L for CuO-NPs, confirming that the nanoparticulate form of materials plays an important role. At 100 mg/L and 200 mg/L, Zn exhibited significantly different effects between the bulk and nano treatments.

**Fig. 6:**
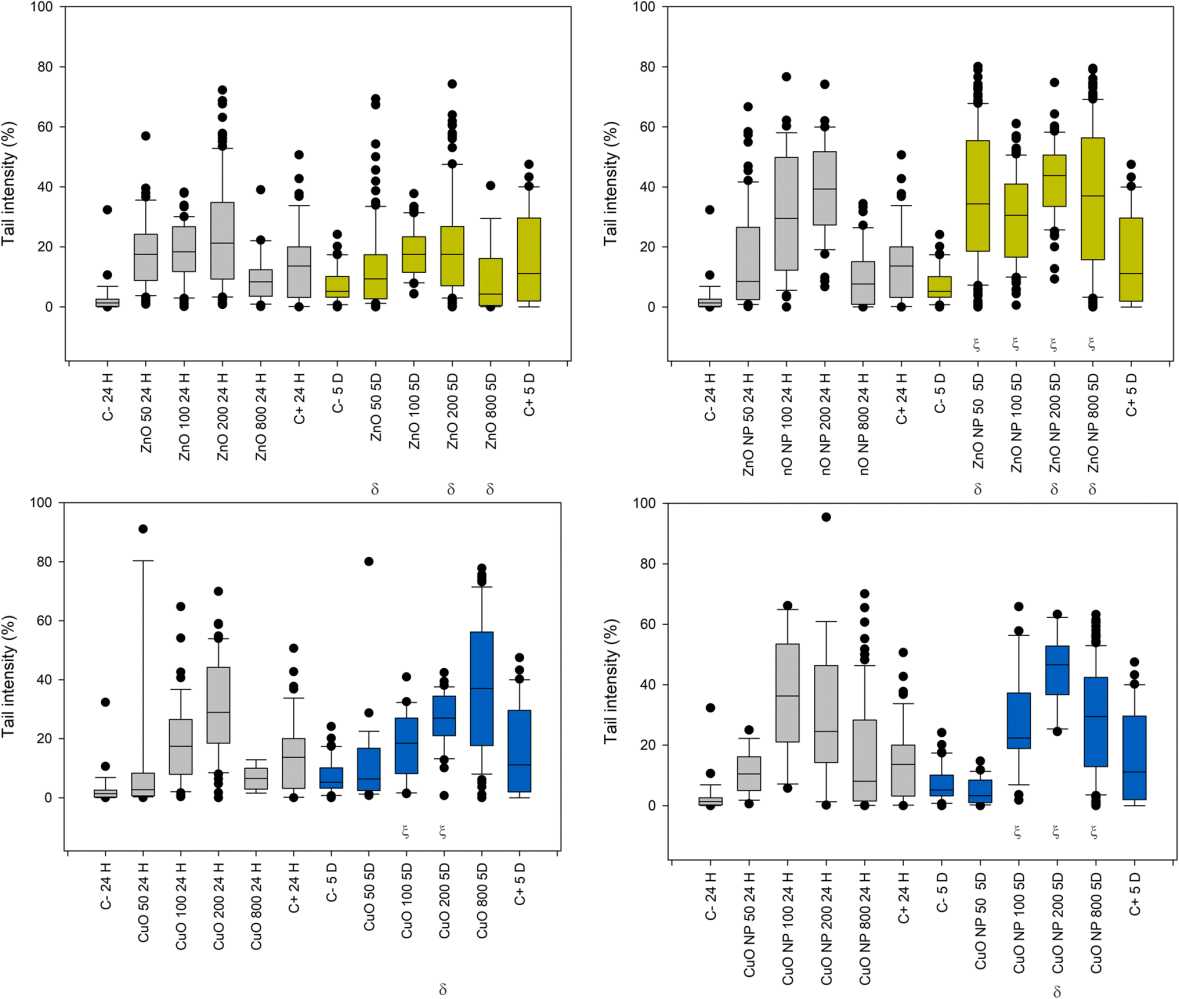
Box plots for the distribution of tail intensity results in *R. communis* exposed to ZnO and CuO (bulk and nano forms) after 24 h and 5 days of treatment. The symbol ξ indicates a significant difference against negative control (C-), within treatment and time. δ indicates differences between treatments within concentration and time

Tail intensity results, shown in Fig. 6, are of significant relevance. The three-way ANOVA reports a triple interaction between factors for both ZnO and CuO pair of treatments (bulk and nano forms). Tail intensity, which showed better sensitivity for this research, reports significant differences against the negative control for all the tested concentrations of ZnO-NPs, for the CuO 200 and 800 mg/L at 24 h, and for the three higher concentrations of CuO-NPs at 5 days of treatment for both cases. The difference in the effect of the nanoparticulate form is more evident for ZnO-NPs 5 days post-treatment, as there was a significant difference between treatments for 50, 200, and 800 mg/L; the significant difference between CuO treatments (nano vs. bulk) was found only at 200 mg/L after 5 days. These findings underscore the relevance of our research in toxicology and nanotechnology.

**Fig. 7:**
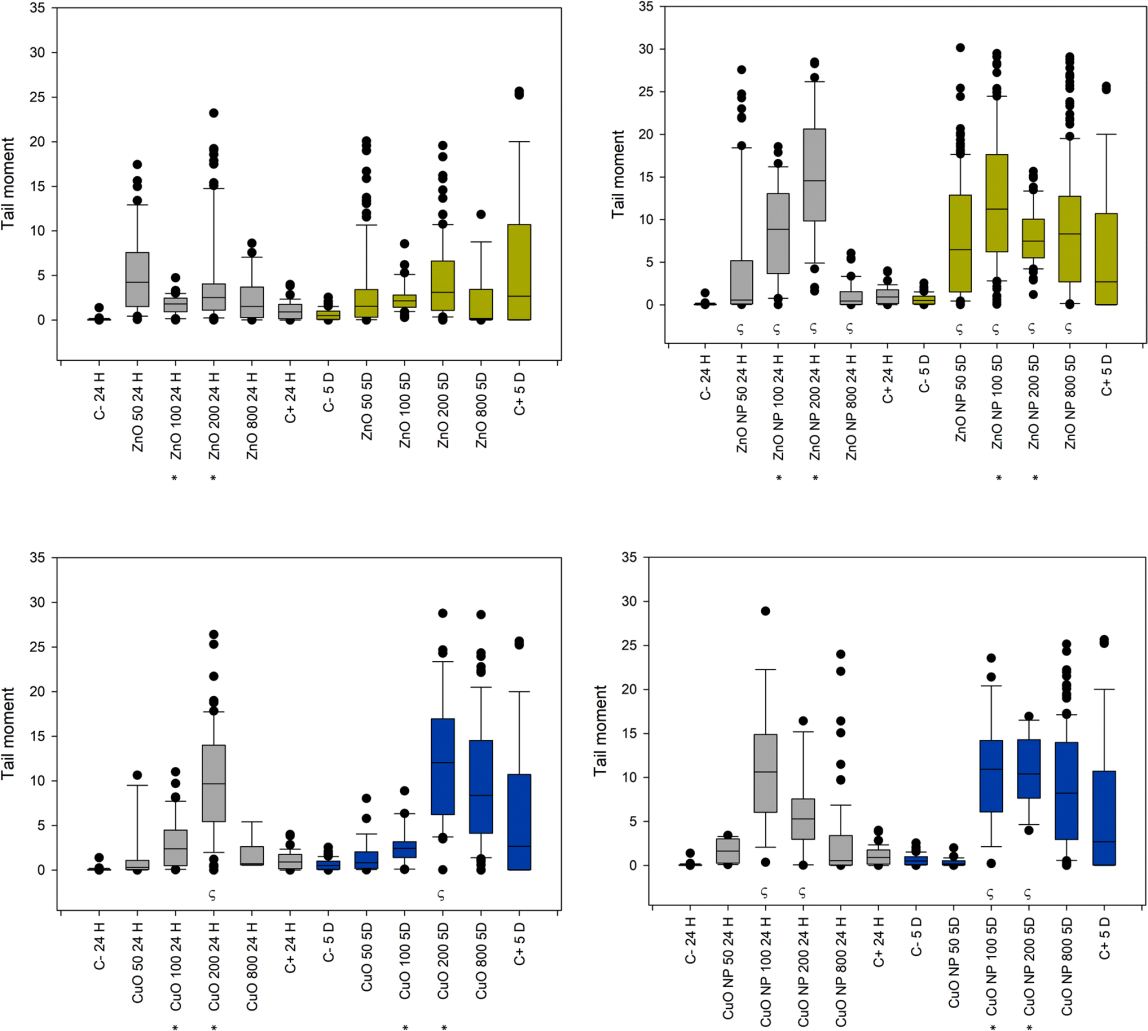
Box plots for the distribution of tail moment results in R. communis exposed to ZnO and CuO (bulk and nano forms) after 24 hours and 5 days of treatment. (C-) negative control (C-), (C+) positive control (C+). * Indicates significant difference against negative control, without considering time. * Indicates a significant difference between bulk and nano treatments within concentration without considering time

Figure 7 shows the box plots of the tail moment, grouped by type of treatment. Three-way ANOVA reported significant interaction between treatments and concentrations, as well as concentration and time for both ZnO and CuO treatments. Data showed no triple interaction between factors. The effect in the tail moment for ZnO-NPs presented a statistically significant difference against negative control for all the evaluated concentrations, suggesting higher toxicity than ZnO-Bulk which presented no statistically significant difference against negative control for any of the evaluated concentrations. The ANOVA results of CuO treatments suggest lower sensitivity to CuO-NPs, as the statistically significant difference against negative control was presented only by 100 mg/L and 200 mg/L. However, it is interesting to note that even CuO-bulk presented a significant difference at a concentration of 200 mg/L. Comparisons among concentrations and treatments at specific times are not possible since there was no triple interaction of factors as reported by ANOVA. The effects of Zn between bulk and nano treatments presented significant differences at concentrations of 100 mg/L and 200 mg/L, confirming the significant contribution of the nanoparticulate form of the material to DNA damage against its bulk counterpart; the same case is presented between CuO bulk and nano treatments at the same concentrations. This may confirm the higher stability of the nanoparticulate form at these two specific concentrations.

All the results obtained by the comet assay show a higher response to concentrations of 100 and 200 mg/L than the results obtained for 800 mg/L. This difference may be due to the instability of highly concentrated nanoparticle dispersions leading to agglomeration and precipitation before uptake. The significant differences found between nano and bulk forms of treatments confirm the potential damage caused by the pollution of nanostructured materials and the importance of performing further studies. These findings show the significant effect of nanoparticle stability and concentration on plant health and the environment, highlighting the importance of our research in this field.

A comprehensive examination of the genotoxic effects of CuO nanoparticles indicated that this increased DNA strand breaks, exhibiting a higher level of damage compared to their bulk counterpart. Moreover, it was observed that DNA damage increased in a concentration-dependent manner until reaching a threshold, beyond which the genotoxicity decreased, possibly due to nanoparticle aggregation, which would reduce their bioavailability. Additionally, DNA damage intensified over time following exposure to the treatment. Previous studies using RAPD analysis have reported that CuO nanoparticles can induce genetic damage in various plant models, such as *Cucumis sativus* and *Hordeum vulgare* (Mosa et al. 2018; Petrova et al. 2021). Similarly, in *Vicia pannonica*, chromosomal aberration assays have demonstrated that these nanoparticles can cause chromosomal breaks and bridge formations (Tasar 2024); these findings may correlate with the comet assay results observed in the present study. Given that copper can participate in Fenton-type reactions and generate reactive oxygen species, it is plausible that the observed damage is, at least in part, the result of oxidative stress on the DNA (Fidalgo et al. 2013). Collectively, these results support the genotoxic potential of CuO nanoparticles.

Besides, ZnO-NPs exhibited genotoxic behavior comparable to that observed with copper oxide nanoparticles, evidenced by increased DNA damage and a higher number of strand breaks compared to their bulk form. Genetic damage increased in a concentration-dependent manner, reaching a threshold after which it plateaued. Moreover, this damage intensified significantly five days after exposure. Several studies have documented that ZnO NPs induce micronuclei formation and DNA breaks in plant models such as *Allium cepa*, *Vicia faba*, and *Nicotiana tabacum*. Consistently, ZnO nanoparticles have been shown to cause significantly more genetic damage than their bulk forms, highlighting the increased toxic potential associated with the nanoscale (Ghosh et al. 2016). These implications call for further research and careful consideration using ZnO nanoparticles.

Additionally, RAPD analyses have shown that ZnO NPs compromise DNA integrity (Plaksenkova et al. 2020). While bulk ZnO is involved in the regulation of antioxidant enzymes, its nanoparticle form has been reported to promote the generation of hydrogen peroxide (H₂O₂), which, through Fenton-type reactions, produces reactive oxygen species capable of inducing oxidative DNA damage (Marreito et al. 2017; Belal and Gad 2023). These findings confirm that ZnO nanoparticles have a higher genotoxic potential than their bulk counterparts, likely mediated by mechanisms related to oxidative stress.

### SEM micrographs

**Fig. 8:**
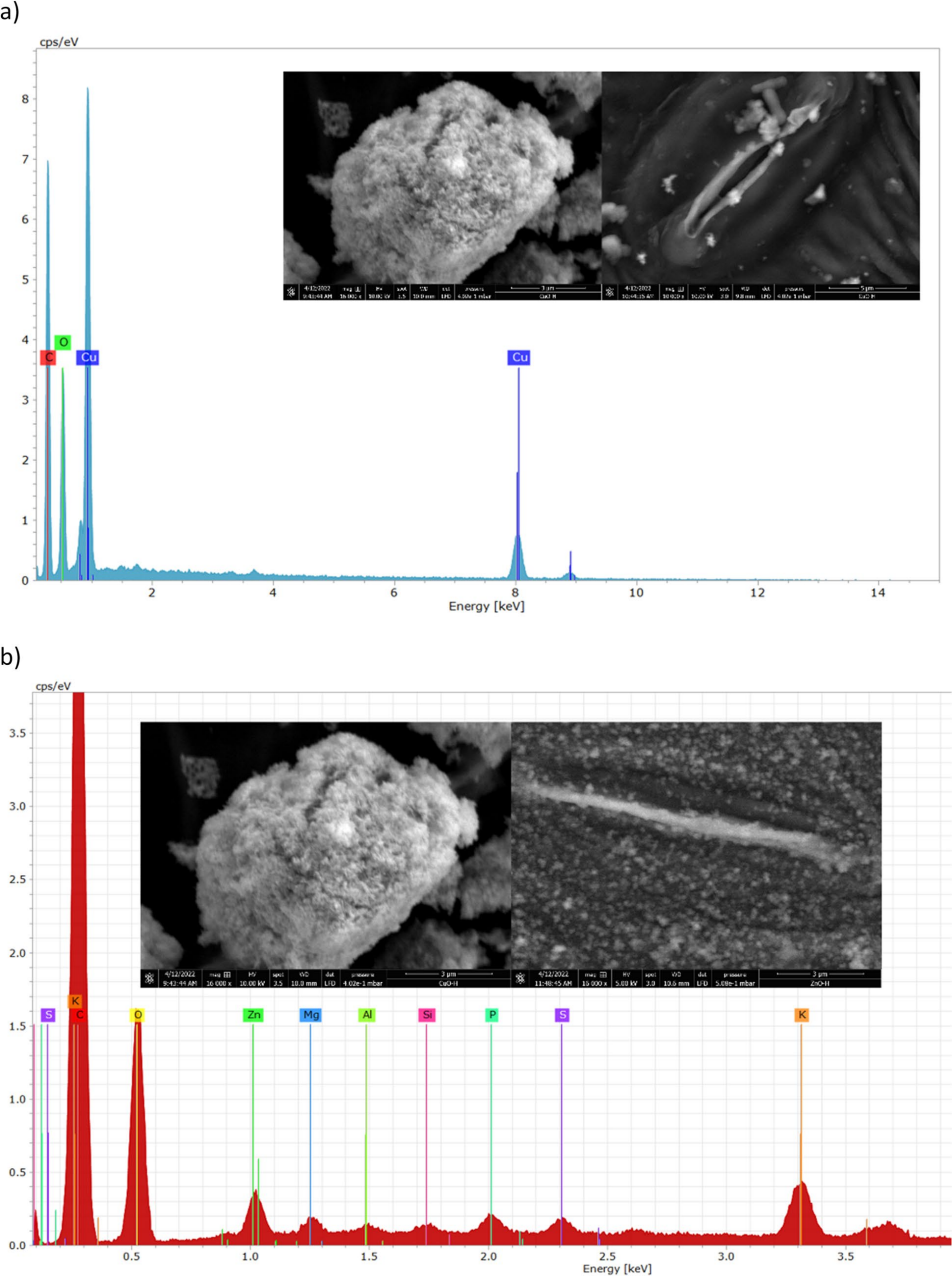
Elemental analysis and SEM micrographs (insets) of ZnO and CuO nanoparticles accumulated inside the stomata

Nanoparticles were observed on the surface of *R. communis* leaves (Fig. 8) for both CuO-NPs (Fig. 8a inset) and ZnO-NPs (Fig. 8b inset). ZnO-NPs exhibited more significant agglomeration on the leaf surface than CuO-NPs, aligning with higher reported toxicity in comet assays. Despite ZnO’s widespread use in fertilizers, this agglomeration may contribute to toxicity as it has been documented to accumulate in leaves and seeds (Siddiqi et al. 2017). Agglomeration near the stomata may restrict their opening upon internalization, a known cause of toxicity (Lv et al. 2019). The presence of Cu and Zn in the accumulated particles near the stomata was confirmed through EDS analysis, potentially inducing internalization. Consistent with prior research, nanoparticle internalization through stomata may lead to DNA damage (Uzu et al. 2010; Zhao et al. 2017; Večeřová et al. 2019). The Cu mass percentage at the point shown in Fig. 8a was 27.9%, while the Zn mass percentage measured at the point shown in Fig. 8b was 1.4%. The remaining contents mainly comprised C and O from the organic composition of samples, the low-working vacuum, and the mounting tape. EDS spectra for leaves exposed to the negative control revealed Cu mass percentages below 0.1% and Zn below 0.01%, likely representing natural plant micronutrients. In all cases, other elements (F, Ca, Cl, K, Mg) were present at very low percentages, with high percentages of Si due to soil dust and sand sediments. EDS analysis of samples exposed to bulk materials showed lower Cu and Zn contents, approximately 1.46% and 0.11%, respectively.

Stomata, essential components of plant physiology, play a crucial role as highly sensitive structures in response to stress caused by exposure to heavy metals. The accumulation of these elements, facilitated by biogeochemical cycles, induces significant alterations in stomatal morphology and functionality, directly impacting vital physiological processes such as transpiration and gas exchange (Guo et al. 2023). Our research, along with various studies, have shown that in environments with high concentrations of heavy metals, these elements tend to deposit on the leaf surface, from where they can penetrate the plant interior through the stomata and subsequently accumulate in different plant tissues (Salih and Aziz 2019). This dynamic is consistent with the observations in Fig. 8, which shows an accumulation of nanoparticles near the stomata, suggesting a potential internalization process that could contribute to the genotoxic damage detected, as indicated by the DNA alterations observed. Furthermore, the same figure clearly illustrates that the stomata begin to exhibit signs of partial or complete closure. This phenomenon is closely linked to an increase in the concentration of abscisic acid (ABA), a key phytohormone known as the “stress hormone.” Under adverse conditions, such as exposure to and internalization of heavy metals, the accumulation of ABA promotes turgor loss in guard cells, inducing stomatal closure as an adaptive strategy to reduce water loss (Xiong et al. 2019). These findings open intriguing possibilities for further research and potential applications in plant physiology and environmental science.

### Spectrophotometry analysis

**Fig. 9:**
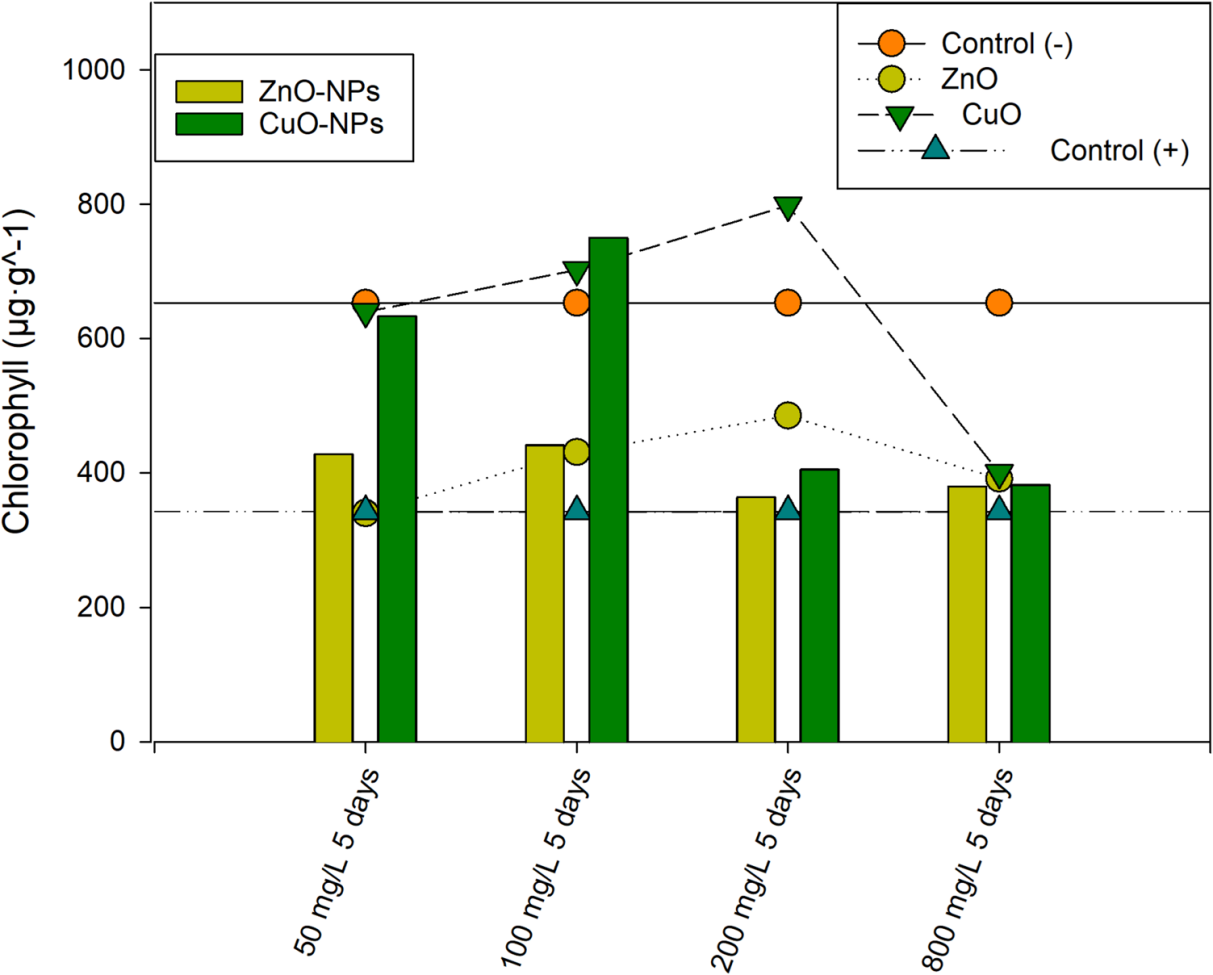
Total chlorophyll. Bars: ZnO-NPs and CuO-NPs. Scatter-lines: Controls and bulk treatments

Figure 9 summarizes chlorophyll quantification 5 days post-plant exposure. ZnO-NPs induced severe chlorosis at 200 mg/L, aligning with maximum DNA damage results reported by comet assay compared to CuO-NP treatment. Two-way ANOVA revealed a significant interaction between concentration and treatment for ZnO vs. ZnO-NPs and CuO vs. CuO-NPs. All concentrations differed significantly from negative and positive controls within each treatment. For ZnO-NPs, chlorosis was similar across concentrations, except for controls and 50 mg/L vs. 100 mg/L, and 50 mg/L vs. 800 mg/L. The DNA damage induced by NPs likely reduces chlorophyll synthesis and structural gene expression for photosystem I, impacting photosynthesis efficiency. Metal ion complexation with phytochelatins may disrupt metal uptake and cellular metal homeostasis pathways (Adhikari et al. 2020). In the case of CuO-NPs, high chlorosis at the highest concentration may result from Cu-induced chemical chlorophyll degradation (Zhao et al. 2017). Considering the valuable insights from chlorophyll content and genotoxic effects, further studies should assess carotenoid levels as important reactive oxygen species scavengers (Adhikari et al. 2020). The pronounced chlorophyll decline induced by CuO-NPs and ZnO-NPs may also be linked to thylakoid stacking reduction and thylakoid membrane damage (Da Costa et al. 2020).

Previous research suggests that nanoparticle stability can affect their toxicity in plants. Aggregated and unstable nanoparticles may cause local effects, such as physical obstruction of the xylem or alteration of the root microbiome (Buzea and Pacheco 2017), while smaller and more stable nanoparticles may have a higher potential for uptake and translocation in plant tissues, resulting in systemic toxicity that affects plant growth and development (Zhu et al. 2008; Wang et al. 2011; Larue et al. 2012a, b). Our study, which involved nebulized nanoparticles that primarily reached the plant leaves, supports these findings. The results provide insight into the effects of nanoparticle bioaccumulation in leaves and highlight the need for further investigation into the mechanisms underlying nanoparticle toxicity in plants, which are not yet fully understood.

The results reflect the genotoxic effects induced by acute exposures and represent a starting point for designing new experimental strategies for a more comprehensive environmental risk assessment. While this type of acute testing constitutes a fundamental tool in regulatory toxicology, oriented toward protection of human health and the environment, the growing concern over the chronic toxic effects of chemical compounds highlights the pressing need to adopt more sustainable and preventive approaches. Continuous or repeated exposure to low levels of pollutants in environmental matrices can have cumulative consequences, affecting both non-target organisms and the stability of ecosystems. In this context, it is essential that genotoxicity assays expand their applicability in ecological risk assessment by incorporating the ability to detect and characterize genotoxic effects resulting from prolonged exposures, which may last for months or even years, depending on the characteristics of the biomonitor used (Macko et al. 2021).

## Conclusions

Comet assay was demonstrated to be sensitive in detecting the genotoxic effect of ZnO-NPs and CuO-NPs, finding significant differences where the rest of the tests were not sensitive enough. LV-SEM allowed the detection of bioaccumulated nanomaterials near the stomata, suggesting the probable internalization through leaves as one of the main ways of toxicity. These findings validate *R. communis* as a bioindicator for genotoxicity caused by nebulized ZnO-NPs and CuO-NPs. The tested concentrations that induced significant DNA damage can be a reference for future studies involving other metal oxide nanoparticles and their interaction with leaf surfaces, particularly concerning stomatal accumulation, which plays a crucial role in nanoparticle toxicity. The comet assay demonstrated significant differences among most tested concentrations, highlighting its sensitivity in evaluating nanoparticle toxicity with plants. The tail length, tail intensity, and tail moment parameters should all be considered, offering valuable information that depends on bioindicator sensitivity and material characteristics.

*R. communis* presented high resistance to the effects of ZnO-NPs and CuO-NPs as reported by total chlorophyll assessment, presenting higher tolerance to CuO treatments in its nano and bulk form. The chlorosis results indicated substantial damage to photosynthesis efficiency by exposure to ZnO-NPs compared to the negative control. The high chlorosis of CuO-NPs and CuO-bulk at 800 mg/L exhibited the most significant chlorosis, possibly due to direct chlorophyll degradation via CuO interaction. Concentration-dependent chlorosis is evident, with important ecotoxicological implications.

Notably, DNA damage induced by lower concentrations of CuO-NP and ZnO-NP increased over time. This finding emphasizes the necessity for extended studies on the genotoxic effects of metal oxide nanoparticles, employing different bioindicators and accounting for potential species-specific tolerance variations to various particle types.

Given that nanomaterials exhibit unique physicochemical properties, such as dynamic agglomeration, a high surface-to-volume ratio, prolonged environmental persistence, and the ability to translocate between biological compartments and tissues, it is crucial to develop and implement standardized toxicological assessment protocols that take these distinct behaviors into account. Conventional toxicology, based on paradigms designed for macro- or molecular-scale chemical compounds, is not always suitable for predicting the potential effects of nanoparticles on organisms and ecosystems. Therefore, only through methodological approaches adapted to the nanoscale—which integrate both acute and chronic studies, as well as in vitro, in vivo, and environmental simulation models—will it be possible to robustly and realistically assess the emerging risks associated with the increasing use of nanomaterials in natural and biological systems. This adaptation is essential to ensure responsible and informed management of nanotechnological development, aligning with sustainability and environmental protection principles, and to mitigate the potentially severe risks that come with the increasing use of nanomaterials.

## Data Availability

The datasets used and analyzed during the current study are available from the corresponding author upon reasonable request.

## References

[CR1] Abdul W, Hajrah N, Sabir-Jamal SM, Al-Garni S, Sabir M, Kabli S, Saini K, Bora R (2018) Therapeutic role of Ricinus communis L. and its bioactive compounds in disease prevention and treatment. Asian Pac J Trop Med 11(3):177. 10.4103/1995-7645.228431

[CR2] Abrica-González P, Zamora-Justo JA, Chavez-Sandoval BE, Vázquez-Martínez GR, Balderas-López JA (2018) Measurement of the optical properties of gold colloids by photoacoustic spectroscopy. Int J Thermophys 39(8):1–7. 10.1007/s10765-018-2412-1

[CR3] Abrica-González P, Gómez-Arroyo S, Jazcilevich-Diamant A, Sotelo-López A, Flores-Márquez AR, Cortés-Eslava J (2023) Evaluation of toxicological effects of ZnO and CuO nanoparticles with Taraxacum officinale as bioindicator. Water Air Soil Pollut 234(7):443. 10.1007/s11270-023-06432-3

[CR4] Adhikari S, Adhikari A, Ghosh S, Roy D, Azahar I, Basuli D, Hossain Z (2020) Assessment of ZnO-NPs toxicity in maize: an integrative microRNAomic approach. Chemosphere 249:126197. 10.1016/j.chemosphere.2020.126197 32087455 10.1016/j.chemosphere.2020.126197

[CR5] Asif N, Malik M, Chaudhry FN (2018) A review of environmental pollution bioindicators. Pollution 4 (1):111–118. 10.22059/poll.2017.237440.296

[CR6] Baker AJM, Reeves RD, Hajar ASM (1994) Heavy metal accumulation and tolerance in British populations of the metallophyte Thlaspi caerulescens J. & C. Presl (Brassicaceae). New Phytol 127(1):61–68. 10.1111/j.1469-8137.1994.tb04259.x 33874394 10.1111/j.1469-8137.1994.tb04259.x

[CR7] Belal R, Gad A (2023) Zinc oxide nanoparticles induce oxidative stress, genotoxicity, and apoptosis in the hemocytes of *Bombyx mori* larvae. Sci Rep 13(1):3520. 10.1038/s41598-023-30444-y 36864109 10.1038/s41598-023-30444-y PMC9981692

[CR8] Buzea C, Pacheco I (2017) Nanomaterials and their classification. In: Shukla A (eds) EMR/ESR/EPR Spectroscopy for characterization of nanomaterials. Adv Struct Mater, Springer, New Delhi, 63 pp 3–45. 10.1007/978-81-322-3655-9_1

[CR9] Calderón-Garcidueñas L, Ayala A (2022) Air Pollution, ultrafine particles, and your brain: are combustion nanoparticle emissions and engineered nanoparticles causing preventable fatal neurodegenerative diseases and common neuropsychiatric outcomes? Environ Sci Technol 56(11):6847–6856. 10.1021/acs.est.1c04706 35193357 10.1021/acs.est.1c04706

[CR10] Collins A, Møller P, Gajski G, Vodenková S, Abdulwahed A, Anderson D, Bankoglu EE, Bonassi S, Boutet-Robinet E, Brunborg G, Chao C, Cooke MS, Costa C, Costa S, Dhawan A, de Lapuente J, Bo’ CD, Dubus J, Dusinska M, Duthie SJ, Yamani NE, Engelward B, Gaivão I, Giovannelli L, Godschalk R, Guilherme S, Gutzkow KB, Habas K, Hernández A, Herrero O, Isidori M, Jha AN, Knasmüller S, Kooter IM, Koppen G, Kruszewski M, Ladeira C, Laffon B, Larramendy M, Hégarat LL, Lewies A, Lewinska A, Liwszyc GE, de Cerain AL, Manjanatha M, Marcos R, Milić M, de Andrade VM, Moretti M, Muruzabal D, Novak M, Oliveira R, Olsen AK, Owiti N, Pacheco M, Pandey AK, Pfuhler S, Pourrut B, Reisinger K, Rojas E, Rundén-Pran E, Sanz-Serrano J, Shaposhnikov S, Sipinen V, Smeets K, Stopper H, Teixeira JP, Valdiglesias V, Valverde M, van Acker F, van Schooten FJ, Vasquez M, Wentzel JF, Wnuk M, Wouters A, Žegura B, Zikmund T, Langie SAS, Azqueta A (2023) Measuring DNA modifications with the comet assay: a compendium of protocols. Nat Protoc 18:929–989. 10.1038/s41596-022-00754-y 36707722 10.1038/s41596-022-00754-y PMC10281087

[CR11] Cortés-Eslava J, Gómez-Arroyo S, Risueño MC, Testillano PS (2018) The effects of organophosphorus insecticides and heavy metals on DNA damage and programmed cell death in two plant models. Environ Pollut 240:77–86. 10.1016/j.envpol.2018.04.119 29729572 10.1016/j.envpol.2018.04.119

[CR12] Da Costa MVJ, Kevat N, Sharma PK (2020) Copper oxide nanoparticle and copper (II) ion exposure in *Oryza sativa* reveals two different mechanisms of toxicity. Water Air Soil Poll 231(6):258. 10.1007/s11270-020-04592-0

[CR13] Dailey LA, Schmehl T, Gessler T, Wittmar M, Grimminger F, Seeger W, Kissel T (2003) Nebulization of biodegradable nanoparticles: impact of nebulizer technology and nanoparticle characteristics on aerosol features. J Control Release 86(1):131–144. 10.1016/S0168-3659(02)00370-X 12490379 10.1016/s0168-3659(02)00370-x

[CR14] Dong B, Ikonnikova I, Rogulin R, Sakulyeva T, Mikhaylov A (2021) Environmental-economic approach to optimization of transport communication in megacities. J Environ Sci Heal A 56(6):660–666. 10.1080/10934529.2021.1913928 10.1080/10934529.2021.1913928 34162313

[CR15] Fidalgo F, Azenha M, Silva AF, Sousa A, Santiago A, Ferraz P, Teixeira J (2013) Copper-induced stress in *Solanum nigrum L.* and antioxidant defense system responses. Food Energy Secur 2:70–80. 10.1002/fes3.20

[CR16] Ghosh M, Jana A, Sinha S, Jothiramajayam M, Nag A, Chakraborty A, Mukherjee A, Mukherjee A (2016) Effects of ZnO nanoparticles in plants: cytotoxicity, genotoxicity, deregulation of antioxidant defenses, and cell-cycle arrest. Mutat Res Genet Toxicol Environ Mutagen 807:25–32. 10.1016/j.mrgentox.2016.07.006 27542712 10.1016/j.mrgentox.2016.07.006

[CR17] Gichner T (2003) Differential genotoxicity of ethyl methanesulphonate, N-ethyl-N-nitrosourea and maleic hydrazide in *tobacco seedlings* based on data of the comet assay and two recombination assays. Mutat Res 538:171–179. 10.1016/S1383-5718(03)00117-7 12834766 10.1016/s1383-5718(03)00117-7

[CR18] Gichner T, Plewa MJ (1998) Induction of somatic DNA damage as measured by single cell gel electrophoresis and point mutation in leaves of tobacco plants. Mutat Res 401:143–152. 10.1016/s0027-5107(98)00003-7 9639693 10.1016/s0027-5107(98)00003-7

[CR19] Guo Z, Gao Y, Yuan X, Yuan M, Huang L, Wang S, Liu C, Duan C (2023) Effects of heavy metals on stomata in plants: a review. Int J Mol Sci 24(11):9302. 10.3390/ijms24119302 37298252 10.3390/ijms24119302 PMC10252879

[CR20] Hou J, Zhou Y, Wang C, Li S, Wang X (2017) Toxic effects and molecular mechanism of different types of silver nanoparticles to the aquatic crustacean *Daphnia magna*. Environ Sci Technol 51(21):12868–12878. 10.1021/acs.est.7b03918 28968066 10.1021/acs.est.7b03918

[CR21] Ibrahim RK, Hayyan M, AlSaadi MA, Hayyan A, Ibrahim S (2016) Environmental application of nanotechnology: air, soil, and water. Environ Sci Pollut Res 23(14):13754–13788. 10.1007/s11356-016-6457-z 10.1007/s11356-016-6457-z 27074929

[CR22] Khalid N, Masood A, Noman A, Aqeel M, Qasim M (2019) Study of the responses of two biomonitor plant species (*Datura alba* & *Ricinus communis*) to roadside air pollution. Chemosphere 235:832–841. 10.1016/j.chemosphere.2019.06.143 31284131 10.1016/j.chemosphere.2019.06.143

[CR23] Koppen G, Azqueta A, Pourrut B, Brunborg G, Collins AR, Langie SAS (2017) The next three decades of the comet assay: a report of the 11th International comet assay workshop. Mutagenesis 32(3):397–408. 10.1093/mutage/gex002 28340065 10.1093/mutage/gex002

[CR24] Larue C, Laurette J, Herlin-Boime N, Khodja H, Fayard B, Flank AM, Brisset F, Carriere M (2012a) Accumulation, translocation and impact of TiO2 nanoparticles in wheat (*Triticum aestivum* spp.): influence of diameter and crystal phase. Sci Total Environ 431:197–208. 10.1016/j.scitotenv.2012.04.073 22684121 10.1016/j.scitotenv.2012.04.073

[CR25] Larue C, Pinault M, Czarny B, Georgin D, Jaillard D, Bendiab N, Mayne-L’Hermite M, Taran F, Dive V, Carrière M (2012b) Quantitative evaluation of multi-walled carbon nanotube uptake in wheat and rapeseed. J Hazard Mater 227–228:155–163. 10.1016/j.jhazmat.2012.05.033 10.1016/j.jhazmat.2012.05.033 22652322

[CR26] Lee CW, Mahendra S, Zodrow K, Li D, Tsai Y, Braam J, Alvarez PJJ (2010) Developmental phytotoxicity of metal oxide nanoparticles to Arabidopsis thaliana. Environ Toxicol Chem 29(3):669–675. 10.1002/etc.58 20821493 10.1002/etc.58

[CR27] Lomash V, Parihar SK, Jain NK, Katiyar AK (2010) Effect of *Solanum nigrum* and *Ricinus communis* extracts on histamine and carrageenan-induced inflammation in the chicken skin. Cell Mol Biol 56(3):1239-1251. 10.1170/141 20158977

[CR28] Lv J, Christie P, Zhang S (2019) Uptake, translocation, and transformation of metal-based nanoparticles in plants: recent advances and methodological challenges. Environ Sci Nano 6(1):41–59. 10.1039/C8EN00645H

[CR29] Macko P, Palosaari T, Whelan M (2021) Extrapolating from acute to chronic toxicity in vitro. Toxicol Vitro 76:105206. 10.1016/j.tiv.2021.105206 10.1016/j.tiv.2021.105206 PMC8434427 34186185

[CR30] Marreiro DD, Cruz KJ, Morais JB, Beserra JB, Severo JS, de Oliveira AR (2017) Zinc and oxidative stress: current mechanisms. Antioxidants (Basel) 6(2):24. 10.3390/antiox6020024 28353636 10.3390/antiox6020024 PMC5488004

[CR31] Maynard AD, Aitken RJ, Butz T, Colvin V, Donaldson K, Oberdörster G, Philbert MA, Ryan J, Seaton A, Stone V, Tinkle SS, Tran L, Walker NJ, Warheit DB (2006) Safe handling of nanotechnology. Nature 444(7117):267–269. 10.1038/444267a 17108940 10.1038/444267a

[CR32] Mendes MG, Santos Junior CD, Dias AC, Bonetti AM (2015) Castor bean (Ricinus communis L.) as a potential environmental bioindicator. Genet Mol Res 14(4):12880–12887. 10.4238/2015 26505440 10.4238/2015.October.21.8

[CR33] Miri A, Mahdinejad N, Ebrahimy O, Khatami M, Sarani M (2019) Zinc oxide nanoparticles: biosynthesis, characterization, antifungal and cytotoxic activity. Mater Sci Eng C 104. 10.1016/j.msec.2019.10998 10.1016/j.msec.2019.109981 31500056

[CR34] Misík M, Ma TH, Nersesyan A, Monarca S, Kim JK, Knasmueller S (2011) Micronucleus assays with *Tradescantia* pollen tetrads: an update. Mutagenesis 26(1):215–221. 10.1093/mutage/geq080 21164205 10.1093/mutage/geq080

[CR35] Møller P, Azqueta A, Boutet-Robinet E, Koppen G, Bonassi S, Milić M, Gajski G, Costa S, Teixeira JP, Pereira CC, Dusinska M, Godschalk R, Brunborg G, Gutzkow KB, Giovannelli L, Cooke MS, Richling E, Laffon B, Valdiglesias V, Basaran N, Del Bo’ C, Zegura B, Novak M, Stopper H, Vodicka P, Vodenkova S, de Andrade VM, Sramkova M, Gabelova A, Collins A, Langie SAS (2020) Minimum information for reporting on the comet assay (MIRCA): recommendations for describing comet assay procedures and results. Abstract Nature Protocols 15(12):3817–3826. 10.1038/s41596-020-0398-1 10.1038/s41596-020-0398-1 PMC7688437 33106678

[CR36] Mosa KA, El-Naggar M, Ramamoorthy K, Alawadhi H, Elnaggar A, Wartanian S, Ibrahim E, Hani H (2018) Copper nanoparticles induced genotoxicity, oxidative stress, and changes in superoxide dismutase (SOD) gene expression in cucumber (*Cucumis sativus*) plants. Front Plant Sci 9:872. 10.3389/fpls.2018.00872 30061904 10.3389/fpls.2018.00872 PMC6055047

[CR37] Mošenoka A, Kokina I, Plaksenkova I, Jermaļonoka M, Sledevskis E, Krasovska M (2024) Effects of metal oxide nanoparticles on the growth and genotoxicity of garden cress (Lepidium sativum L). Agronomy 14(10):2324. 10.3390/agronomy14102324

[CR38] Muthusaravanan S, Sivarajasekar N, Vivek JS, Paramasivan T, Naushad M, Prakashmaran J, Gayathri V, Al-Duaij OK (2018) Phytoremediation of heavy metals: mechanisms, methods and enhancements. Environ Chem Lett 16(4):1339–1359. 10.1007/s10311-018-0762-3

[CR39] Nabila MI, Kannabiran K (2018) Biosynthesis, characterization and antibacterial activity of copper oxide nanoparticles (CuO NPs) from actinomycetes. Biocatal Agric Biotechnol 15:56–62. 10.1016/j.bcab.2018.05.011

[CR40] Navada KM, Nagaraja GK, D’Souza JN, Kouser S, Ranjitha R, Manasa DJ (2020) Phyto assisted synthesis and characterization of Scoparia dulsis L. leaf extract mediated porous nano CuO photocatalysts and its anticancer behavior. Appl Nanosci (Switzerland) 10(11):4221–4240. 10.1007/s13204-020-01536-2

[CR41] Petrova A, Plaksenkova I, Kokina I, Jermaļonoka M (2021) Effect of Fe3O4 and CuO nanoparticles on morphology, genotoxicity, and miRNA expression on different barley (Hordeum vulgare L.) genotypes. Sci World J 2021:6644689. 10.1155/2021/6644689 10.1155/2021/6644689 PMC7884165 33628139

[CR42] Plaksenkova I, Kokina I, Petrova A, Jermaļonoka M, Gerbreders V, Krasovska M (2020) The impact of zinc oxide nanoparticles on cytotoxicity, genotoxicity, and miRNA expression in barley (Hordeum vulgare L.) seedlings. Sci World J 2020:6649746. 10.1155/2020/6649746 10.1155/2020/6649746 PMC7725555 33343237

[CR43] Rabajczyk A, Zielecka M, Porowski R, Hopke PK (2020) Metal nanoparticles in the air: state of the art and future perspectives. Environ Sci Nano 7:3233–3254. 10.1039/D0EN00536C

[CR44] Rai PK (2016) Impacts of particulate matter pollution on plants: implications for environmental biomonitoring. Ecotoxicol Environ Saf 129:120–136. 10.1016/j.ecoenv.2016.03.012 27011112 10.1016/j.ecoenv.2016.03.012

[CR45] Raklami A, Oubane M, Meddich A, Hafidi M, Marschner B, Heinze S, Oufdou K (2021) Phytotoxicity and genotoxicity as a new approach to assess heavy metals effect on Medicago sativa L.: role of metallo-resistant rhizobacteria. Environ Technol Innov 24:101833. 10.1016/j.eti.2021.101833

[CR46] Rotini A, Tornambè A, Cossi R, Iamunno F, Benvenuto G, Berducci MT, Maggi C, Thaller MC, Cicero AM, Manfra L, Migliore L (2017) Salinity-based toxicity of CuO nanoparticles, CuO-bulk and Cu ion to *Vibrio anguillarum*. Front Microbiol 8:2076. 10.3389/fmicb.2017.02076 29118743 10.3389/fmicb.2017.02076 PMC5661029

[CR47] Salih Z, Aziz F (2019) Heavy metals accumulation in leaves of five plant species as a bioindicator of steel factory pollution and their effects on pigment content. Pol J Environ Stud 28(6):4351–4358. 10.15244/pjoes/99304

[CR48] Sharaf-Zeebaree SY, Sharaf-Zeebaree AY, Haji-Zebari OI, Sharaf-Zebari AY (2021) Sustainable fabrication, optical properties and rapid performance of bio-engineered copper nanoparticles in removal of toxic methylene blue dye in an aqueous medium. Curr Res Green Sustain Chem 4:100103. 10.1016/j.crgsc.2021.100103

[CR49] Siddiq KS, Husen A (2017) Plant response to engineered metal oxide nanoparticles. Nanoscale Res Lett 12(1):92. 10.1186/s11671-017-1861-y 28168616 10.1186/s11671-017-1861-y PMC5293712

[CR50] Singh NP, McCoy MT, Tice RR, Schneider LE (1988) A simple technique for quantitation of low levels of DNA damage in individual cells. Exp Cell Res 175:184–191. 10.1016/0014-4827(88)90265-0 3345800 10.1016/0014-4827(88)90265-0

[CR51] Slomberg DL, Schoenfisch MH (2012) Silica nanoparticle phytotoxicity to Arabidopsis thaliana. Environ Sci Technol 46(18):10247–10254. 10.1155/2021/664468910.1021/es300949f 22889047 10.1021/es300949f

[CR52] de Souza-Costa ET, Guilherme LRG, de Melo ÉEC, Ribeiro BT, dos Santos IE, da Costa-Severiano E, Faquin V, Hale BA (2012) Assessing the tolerance of castor bean to Cd and Pb for phytoremediation purposes. Biol Trace Elem Res 145(1):93–100. 10.1007/s12011-011-9164-0 21826609 10.1007/s12011-011-9164-0

[CR53] Swislowski P, Vergel K, Zinicovscaia I, Rajfur M, Wacławek M (2022) Mosses as a biomonitor to identify elements released into the air as a result of car workshop activities. Ecol Indic 138:108849. 10.1016/j.ecolind.2022.108849

[CR54] Tasar N (2024) Genotoxic effects of copper nanoparticles on Vicia pannonica Crantz. root meristem cells. J Nanopart Res 26:185. 10.1007/s11051-024-06096-y

[CR55] Uzu G, Sobanska S, Sarret G, Muñoz M, Dumat C (2010) Foliar lead uptake by lettuce exposed to atmospheric fallouts. Environ Sci Technol 44(3):1036–1042. 10.1021/es902190u 20063891 10.1021/es902190u

[CR56] Večeřová K, Večeřa Z, Mikuška P, Coufalík P, Oravec M, Dočekal B, Novotná K, Veselá B, Pompeiano A, Urban O (2019) Temperature alters susceptibility of Picea abies seedlings to airborne pollutants: the case of CdO nanoparticles. Environ Pollut 253:646–654. 10.1016/j.envpol.2019.07.061 31330356 10.1016/j.envpol.2019.07.061

[CR57] Wang H, Kou X, Pei Z, Xiao JQ, Shan X, Xing B (2011) Physiological effects of magnetite (Fe3O4) nanoparticles on perennial ryegrass (Lolium perenne L.) and pumpkin (Cucurbita mixta) plants. Nanotoxicology 5(1):30–42. 10.3109/17435390.2010.489206 21417686 10.3109/17435390.2010.489206

[CR58] Xiong T, Zhang T, Dumat C, Sobanska S, Dappe V, Shahid M, Xian Y, Li X, Li S (2019) Airborne foliar transfer of particular metals in *Lactuca sativa* L.: translocation, phytotoxicity, and bioaccessibility. Environ Sci Pollut Res Int 26(20):20064–20078. 10.1007/s11356-018-3084-x 10.1007/s11356-018-3084-x 30178413

[CR59] Yuvarajan D, Dinesh-Babu M, Beem-Kumar N, Amith Kishore P (2018) Experimental investigation on the influence of titanium dioxide nanofluid on emission pattern of biodiesel in a diesel engine. Atmos Pollut Res 9(1):47–52. 10.1016/j.apr.2017.06.003

[CR60] Zhao J, Ren W, Dai Y, Liu L, Wang Z, Yu X, Zhang J, Wang X, Xing B (2017) Uptake, distribution, and transformation of CuO NPs in a floating plant Eichhornia crassipes and related stomatal responses. Environ Sci Technol 51(13):7686–7695. 10.1021/acs.est.7b01602 28586199 10.1021/acs.est.7b01602

[CR61] Zhu H, Han J, Xiao JQ, Jin Y (2008) Uptake, translocation, and accumulation of manufactured iron oxide nanoparticles by pumpkin plants. J Environ Monit 10(6):713. 10.1039/b805998e 18528537 10.1039/b805998e

